# World Society of Emergency Surgery (WSES) guidelines for management of skin and soft tissue infections

**DOI:** 10.1186/1749-7922-9-57

**Published:** 2014-11-18

**Authors:** Massimo Sartelli, Mark A Malangoni, Addison K May, Pierluigi Viale, Lillian S Kao, Fausto Catena, Luca Ansaloni, Ernest E Moore, Fred A Moore, Andrew B Peitzman, Raul Coimbra, Ari Leppaniemi, Yoram Kluger, Walter Biffl, Kaoru Koike, Massimo Girardis, Carlos A Ordonez, Mario Tavola, Miguel Cainzos, Salomone Di Saverio, Gustavo P Fraga, Igor Gerych, Michael D Kelly, Korhan Taviloglu, Imtiaz Wani, Sanjay Marwah, Miklosh Bala, Wagih Ghnnam, Nissar Shaikh, Osvaldo Chiara, Mario Paulo Faro, Gerson Alves Pereira, Carlos Augusto Gomes, Federico Coccolini, Cristian Tranà, Davide Corbella, Pietro Brambillasca, Yunfeng Cui, Helmut A Segovia Lohse, Vladimir Khokha, Kenneth YY Kok, Suk-Kyung Hong, Kuo-Ching Yuan

**Affiliations:** Department of Surgery, Macerata Hospital, Via Santa Lucia 2, Macerata, 62019 Italy; American Board of Surgery, Philadelphia, USA; Division of Trauma and Surgical Critical Care, Vanderbilt University Medical Center, Nashville, Tennessee USA; Clinic of Infectious Diseases, St Orsola-Malpighi University Hospital, Bologna, Italy; Department of Surgery, The University of Texas Medical School, Houston, USA; Emergency Surgery Department, Maggiore Parma Hospital, Parma, Italy; General Surgery I, Papa Giovanni XXIII Hospital, Bergamo, Italy; Department of Surgery, Denver Health Medical Center, Denver, USA; Department of Surgery, University of Florida, Gainesville, Florida USA; Department of Surgery, University of Pittsburgh, Medical Center, Pittsburgh, USA; Department of Surgery, UC San Diego Health System, San Diego, USA; Department of Abdominal Surgery, University Hospital Meilahti, Helsinki, Finland; Department of General Surgery, Rambam Health Care Campus, Haifa, Israel; Department of Primary Care & Emergency Medicine, Kyoto University Graduate School of Medicine, Kyoto, Japan; Intensive Care Unit, University of Modena, Modena, Italy; Department of Surgery, Fundación Valle del Lilí, Universidad del Valle, Cali, Colombia; Department of Anesthesia and ICU, Villa Scazzi Hospital, Genoa, Italy; Department of Surgery, University of Santiago de Compostela, Santiago de Compostela, Spain; Trauma Surgery Unit, Maggiore Hospital, Bologna, Italy; Division of Trauma Surgery, Hospital de Clinicas, School of Medical Sciences, University of Campinas, Campinas, Brazil; Department of Surgery 1, Lviv Regional Hospital, DanyloHalytskyLviv National Medical University, Lviv, Ukraine; Griffith Base Hospital, Griffith, NSW Australia; Department of General Surgery, Istanbul Doctor’s Center, Istanbul, Turkey; Department of Surgery, Sheri-Kashmir Institute of Medical Sciences, Srinagar, India; Department of Surgery, Pt BDS Post-graduate Institute of Medical Sciences, Rohtak, India; General Surgery and Trauma Unit, Hadassah Hebrew University Medical Center, Jerusalem, Israel; Department of Surgery Mansoura, Faculty of medicine, Mansoura University, Mansoura, Egypt; Department of Anesthesia and ICU, Hamad Medical Corporation, Doha, Qatar; Emergency Department, Niguarda Ca’Granda Hospital, Milan, Italy; Department of General and Gastrointestinal Surgery, Trauma and Emergency Surgery Division, ABC Medical School, Santo André, SP Brazil; Emergency Surgery and trauma Unit, Department of Surgery, Ribeirão, Preto Brazil; Hospital Universitário Therezinha de Jesus, Faculdade de Ciências Médicas e da Saúde de Juiz de Fora (SUPREMA), Universidade Federal de Juiz de Fora (UFJF), Minas Gerais, Brasil; Department of Anestesiology, Papa Giovanni XXIII Hospital, Bergamo, Italy; Department of Surgery, Tianjin Nankai Hospital, Nankai Clinical School of Medicine, Tianjin Medical University, Tianjin, China; II Cátedra de Clínica Quirúrgica, Hospital de Clínicas, Universidad Nacional de Asunción, San Lorenzo, Paraguay; Department of Surgery, Mozyr City Hospital, Mozyr, Belarus; Department of Surgery, Ripas Hospital, Bandar Seri Begawan, Brunei; Division of Trauma and Surgical Critical Care, Department of Surgery, University of Ulsan, Seoul, Republic of Korea; Department of Trauma and Emergency Surgery, Chang Gung Memorial Hospital, Taipei, Taiwan

## Abstract

Skin and soft tissue infections (SSTIs) encompass a variety of pathological conditions ranging from simple superficial infections to severe necrotizing soft tissue infections. Necrotizing soft tissue infections (NSTIs) are potentially life-threatening infections of any layer of the soft tissue compartment associated with widespread necrosis and systemic toxicity. Successful management of NSTIs involves prompt recognition, timely surgical debridement or drainage, resuscitation and appropriate antibiotic therapy. A worldwide international panel of experts developed evidence-based guidelines for management of soft tissue infections. The multifaceted nature of these infections has led to a collaboration among surgeons, intensive care and infectious diseases specialists, who have shared these guidelines, implementing clinical practice recommendations.

## Executive summary

Skin and soft tissue infections (SSTIs) encompass a variety of pathological conditions involving the skin and underlying subcutaneous tissue, fascia, or muscle and ranging from simple superficial infections to severe necrotizing infections that may involve the dermal, subcutaneous, fascia, and muscle layers [1].

SSTIs may affect any part of the body, although the lower extremities, the perineum, and the abdominal wall are the most common sites of involvement. SSTIs are a relatively infrequent clinical problem; however, surgeons are often called upon for their management.

These guidelines focus mainly on necrotizing soft tissue infections (NSTIs). The mortality resulting from NSTIs appears to have decreased over the past decade, possibly due to improved recognition and earlier delivery of more effective therapy [2].

Successful management of NSTIs involves prompt recognition, timely surgical debridement or drainage, aggressive resuscitation and appropriate antibiotic therapy.

A worldwide international panel of experts developed evidenced-based guidelines for management of soft tissue infections. The guidelines outline clinical recommendations based on the Grading of Recommendations Assessment, Development, and Evaluation (GRADE) hierarchy criteria summarized in Table 1 [3, 4].

**Table 1:** **Grading of recommendations from Guyatt and colleagues** [3, 4]

Grade of recommendation	Clarity of risk/benefit	Quality of supporting evidence	Implications
1A			
Strong recommendation, high-quality evidence	Benefits clearly outweigh risk and burdens, or vice versa	RCTs without important limitations or overwhelming evidence from observational studies	Strong recommendation, applies to most patients in most circumstances without reservation
1B			
Strong recommendation, moderate-quality evidence	Benefits clearly outweigh risk and burdens, or vice versa	RCTs with important limitations (inconsistent results, methodological flaws, indirect analyses or imprecise conclusions) or exceptionally strong evidence from observational studies	Strong recommendation, applies to most patients in most circumstances without reservation
1C			
Strong recommendation, low-quality or very low-quality evidence	Benefits clearly outweigh risk and burdens, or vice versa	Observational studies or case series	Strong recommendation but subject to change when higher quality evidence becomes available
2A			
Weak recommendation, high-quality evidence	Benefits closely balanced with risks and burden	RCTs without important limitations or overwhelming evidence from observational studies	Weak recommendation, best action may differ depending on the patient, treatment circumstances, or social values
2B			
Weak recommendation, moderate-quality evidence	Benefits closely balanced with risks and burden	RCTs with important limitations (inconsistent results, methodological flaws, indirect or imprecise) or exceptionally strong evidence from observational studies	Weak recommendation, best action may differ depending on the patient, treatment circumstances, or social values
2C			
Weak recommendation, Low-quality or very low-quality evidence	Uncertainty in the estimates of benefits, risks, and burden; benefits, risk, and burden may be closely balanced	Observational studies or case series	Very weak recommendation; alternative treatments may be equally reasonable and merit consideration

### Surgical site infections

1) Surgical site infections require prompt and wide opening of the surgical incision. Antimicrobial therapy is recommended for deep incisional surgical site infections if systemic signs of sepsis are present, if source control is incomplete or in immunocompromised patients (Recommendation 1 C).

2) In patients who have had clean operations, antimicrobial therapy should cover gram-positive organisms; in contrast, in patients who have had procedures on the gastrointestinal or genitourinary tract antimicrobial therapy should cover both gram-positive and gram-negative organisms (Recommendation 1 C).

### Non necrotizing superficial soft tissue infections

3) Erysipelas, and cellulitis are managed by antimicrobial therapy against Gram-positive bacteria (Recommendation 1 C).

4) Lack of clinical response for cellulitis may be due to resistant strains of staphylococcus or streptococcus, or deeper processes, such as necrotizing fasciitis or myonecrosis (Recommendation 1 C).

5) Therapy for community-acquired MRSA should be recommended for patients at risk for CA-MRSA or who do not respond to beta-lactam therapy within 48 to 72 hours (Recommendation 1 C).

6) If a streptococcal toxic shock syndrome is suspected, an antiribosomal agent such as clindamycin or linezolid can be used to reduce exotoxin and superantigen production (Recommendation 1 C).

### Simple abscesses

7) Incision and drainage is the primary treatment for simple abscesses or boils. Antibiotics are not needed (Recommendation 1 C).

### Complex abscesses

8) Complex skin and subcutaneous abscesses are typically well circumscribed and respond to incision and drainage. Antimicrobial therapy is required if systemic signs of sepsis are present, in immunocompromised patients, if source control is incomplete or for the abscesses with significant cellulitis (Recommendation 1 C).

9) Empiric antibiotic therapy should be directed toward the likely pathogens involved.

Therapy for community-acquired MRSA should be recommended for patients at risk for CA-MRSA (Recommendation 1 C).

10) Inadequate resolution should prompt consideration of further drainage, resistant pathogens, or host immune failure (Recommendation 1 C).

### Diagnosis of necrotizing soft tissue infections

11) A rapidly progressive soft tissue infection should be always treated aggressively as a necrotizing soft tissue infection (Recommendation 1 C)

12) Both CT and MRI may be useful for diagnosing necrotizing soft tissue infections. However MRI may be difficult to perform under emergency situations. (Recommendation 2 C).

13) In unstable patients, ultrasound may be useful to differentiate simple cellulitis from necrotizing fasciitis (Recommendation 2 C).

### Management of patients with necrotizing soft tissue infections

14) Patients with severe sepsis or septic shock caused by NSTI require early source control, antimicrobial therapy, and supportive treatment (Recommendation 1 C).

### Source control in patients with necrotizing soft tissue infections

15) Surgical source control must be early and aggressive to halt progression of the inflammatory process caused by NSTI (Recommendation 1 C).

### Hyperbaric oxygen in patients with necrotizing soft tissue infections

16) Although the benefit of adjuvant hyperbaric oxygen (HBO) therapy remains controversial, it may be considered where it is available (Recommendation 2 C).

### Antimicrobial therapy in patients with necrotizing soft tissue infections

17) Early appropriate empiric coverage against suspected pathogens should be initiated, based upon the clinical setting for patients with NSTI (Recommendation 1 C).

18) Patients whose clinical setting or gram stain suggests rapidly progressive infection potentiated by exotoxins from Gram positive pathogens (S. pyogenes, CA-MRSA, Clostridial species), treatment with antimicrobial agents should be combined with antiribosomal agents (clindamycin or linezolid). Patients who present with rapidly progressive infections with gram stains of tissue demonstrating gram negative pathogens (Aeromonas sp., Eikenella, Vibrio sp) should be treated with antiribosomal agents targeting gram negative pathogens (tetracyclines) (Recommendation 1 C).

19) Appropriate empiric coverage against MRSA should be immediately initiated in patients with NSTI (Recommendation 1 C).

20) Since it is impossible to exclude with certainty a polymicrobial necrotizing infection, an aggressive broad-spectrum empiric antimicrobial therapy should initially be selected to cover gram-positive, gram-negative, and anaerobic organisms until culture-specific results and sensitivities are available (Recommendation 1 C).

21) An appropriate de-escalation of antimicrobial therapy is suggested once culture results come back (Recommendation 1 C).

### Supportive treatment in patients with necrotizing soft tissue infections

22) Supportive treatment in managing NSTI must be early and aggressive to halt progression of the inflammatory process (Recommendation 1 A).

### Intravenous immunoglobulins in patients with necrotizing soft tissue infections

23) Intravenous immunoglobulins may be considered in all patients with NSTI and evidence of organ dysfunction (Recommendation 2 C).

### Early nutritional support in patients with necrotizing soft tissue infections

24) Early nutritional support should be established (Recommendation 2 C).

### Classification

Several systems of classification have been used to describe SSTIs. In 1998 The US Food and Drug Administration (FDA) classified SSTIs into two broad categories - uncomplicated and complicated - for the purpose of clinical trials evaluating new antimicrobials for the treatment of SSTIs. Uncomplicated SSTIs included superficial infections such as cellulitis, simple abscesses, impetigo, and furuncles that either require antibiotics alone or in conjunction with surgical incision for drainage of abscess. In contrast, complicated SSTIs involve the deep soft tissues and require significant surgical intervention [5].

The terms “complicated” and “uncomplicated” skin structure infections is still valid and can be useful in describing SSTIs [6].

Uncomplicated SSTIs are at low risk for life- or limb-threatening infection unless they are not properly treated. Patients who have uncomplicated SSTIs can be treated with either empiric antibiotic therapy according to likely pathogen and local resistance patterns or simple surgical drainage.

The practice guidelines of the Infectious Diseases Society of America (IDSA) [7] for the diagnosis and management of skin and soft tissue infections classifies SSTIs into five categories: 1) superficial uncomplicated infection (includes impetigo, erysipelas and cellulitis), 2) necrotizing infections, 3) infections associated with bites and animal contact, 4) surgical site infections and 5) infections in the immunocompromised host.

Eron et al. [8] classified SSTIs according to the severity of local and systemic signs and the presence or absence of comorbid conditions for patients presenting as out-patients to guide the clinical management, treatment, and admission decisions for patients with SSTIs. This system is organized into classes of infection:

Class 1: patients with SSTI, but no signs or symptoms of systemic toxicity or co-morbidities.

Class 2: patients are either systemically unwell with stable co-morbidities or are systemically well, but have a comorbidity (e.g. diabetes, obesity) that may complicate or delay resolution.

Class 3: patients appear toxic and unwell (fever, tachycardia, tachypnea and/or hypotension).

Class 4: patients have sepsis syndrome and life-threatening infection, for example necrotizing fasciitis.

SSTIs may be also classified according to the anatomical tissue layers involved [9].

Superficial infections are located at the epidermal and dermal layers, while cellulitis may extend into the subcutaneous tissue. Deep infections extend below the dermis and may involve the subcutaneous tissue, fascial planes or muscular compartments and present as complex abscesses, fasciitis, or myonecrosis.

SSTIs may also be classified as non-necrotizing or necrotizing soft tissue infections [10]. Non-necrotizing, complicated soft tissue infections typically involve one or both of the superficial layers of the skin (epidermis and dermis) and the subcutaneous tissue, such as complex abscesses, but may occasionally involve deeper structures.

NSTI is an inclusive term intended to describe all infections with a necrotizing component involving any or all the layers of the soft tissue compartment, from the superficial dermis and subcutaneous tissue to the deeper fascia and muscle [11].

NSTIs most commonly involve the muscular fascial layers and warrant prompt, aggressive surgical debridement [6].

NSTIs have also been sub-classified according to the type and number of pathogens initiating the infectious process [12]:

Type 1: polymicrobial infections that typically arise from a chronic, indolent source and spread along fascial planes. This type of infection comprises roughly 85-90% of NSTIs; Type 2: monomicrobial gram positive, aerobic cocci, either Streptococcus species or community-acquired methicillin resistant Staphylococcus aureus (CA-MRSA). Its ill effects are related to both toxin production and rapid growth rate of pathogens. These infections comprise about 10-15% of NSTIs; and Type 3: monomicrobial infections initiated by a variety of virulent gram positive or gram negative bacilli such as Clostridia, Vibrio, Aeromonas, Eikenella, or Bacillus species. These are the most uncommon of NSTIs.

### World Society of Emergency Surgery classification

Treatment decisions for SSTI depend on numerous factors including the severity and depth of infection and the clinical setting.

The WSES expert panel classified soft tissue infections using the classification system illustrated below.

**Surgical Site infections**

Incisional

- Superficial
- Deep

**Non-necrotizing SSTIs**

- Superficial infections (Impetigo, erysipelas, cellulitis)
- Simple abscess, boils and carbuncles
- Complex abscesses

**Necrotizing SSTIs (NSTIs)**

- Necrotizing cellulitis
- Necrotizing fasciitis
- Fournier’s gangrene
- Necrotizing myositis

The first group includes surgical site infections (SSIs). Soft tissue non-surgical site infections are divided into non-necrotizing and necrotizing soft tissue infections.

### Surgical site infections

SSIs represent a separate chapter among the soft tissue infections [13]. They are post-operative infections and because of their multifaceted aspects they are framed into a separate group.

The Center for Disease Control and Prevention (CDC) defined criteria for classification of surgical site infections. SSIs are classified as: superficial incisional infection, deep incisional infection, and organ space infection. Superficial incisional infections are the most common type of surgical site infections [14]. Organ space infections are not soft tissue infections.

The development of an SSI depends on contamination of the wound site at the end of a surgical procedure and specifically relates to the pathogenicity and inoculum of microorganisms present, balanced against the host’s immune response.

Numerous patient-related (endogenous) and process/procedures related (exogenous) risk factors for developing an SSI have been described [15].

Some factors, such as age and gender, are obviously not amenable to changes or improvements. However, addressing other potential factors, such as the nutritional status, smoking, proper use of antibiotics and accurate intraoperative technique, can reduce the likelihood of SSI.

Prophylactic antibiotic administration is an established approach for reducing the risk of SSIs in various fields of elective surgery [16, 17].

### Non-necrotizing SSTIs

Non-necrotizing soft tissue infections include superficial infections, complex abscesses, and infections developing in damaged skin (animal and human bites). If untreated, these can evolve into necrotizing infections.

#### Superficial infections

Superficial infections encompass either superficial spreading infection and inflammation within the epidermis and dermis that may be treated with antibiotics alone or a well circumscribed abscess that may be treated by drainage alone.

Physical examination usually reveals erythema, tenderness, and induration. The majority of superficial skin and soft tissue infections are caused by gram positive bacteria, particularly streptococci and S. aureus. Three common presentations consist of impetigo, erysipelas, and cellulitis. They are managed by antimicrobial therapy against Gram-positive bacteria.

Impetigo is a skin infection that is common throughout the world. It is characterized by discrete purulent lesions nearly always caused by β-haemolytic streptococci and/or S. aureus.

Erysipelas is a fiery red, tender, painful plaque with well-demarcated edges and is commonly caused by streptococcal species, usually S. pyogenes. S. aureus rarely causes erysipelas [18].

Cellulitis is an acute bacterial infection of the dermis and the subcutaneous tissue that most commonly affects the lower extremities, although it can affect other areas. It causes local signs of inflammation, such as warmth, erythema, pain, lymphangitis, and frequently systemic upset with fever and raised white blood cell count [19].

For a simple superficial abscess or boil, incision and drainage is the primary treatment, and antibiotics are not needed [20, 21]. To be considered a simple abscess, induration and erythema should be limited only to a defined area of the abscess and should not extend beyond its borders. Additionally, simple abscesses should not have extension into deeper tissues or multiloculated extension.

#### Complex abscesses

Common sites of origin may be perineal or perianal infections, perirectal abscesses, diabetic foot or lower-extremity ulcerations, traumatic injuries, chronic cutaneous cysts, intravenous drug injection sites, gastrointestinal pathology with perforation, genitourinary pathology, animal bites, and pressure ulcers.

Complicated skin and subcutaneous abscesses are typically well circumscribed and respond to incision and drainage with adjuvant antibiotic therapy.

The cornerstone of treatment is early surgical drainage. Antimicrobial therapy is required perioperatively if systemic signs of sepsis are present, in immunocompromised patients, if source control is incomplete or for the abscesses with significant cellulitis. The initiating pathogens often differ according to the originating site. Aerobic gram-positive pathogens are isolated in most complicated abscesses. Depending on the origin, anaerobes, Enterobacteriaceae, and Clostridium spp. may also be present [1].

Although most cases can be managed by incision and drainage, abscesses in injecting drug users (IDUs) require special considerations as compared to soft tissue infections which are not caused by intravenous drug abuse [22–25]. There are two main sources of organisms: the IDUs themselves (their oropharynx, skin or faeces), and the environment. Contamination may occur when the user prepares or injects the drug, uses shared needles or re-uses injection paraphernalia. Manufacturing and handling of injectable drugs may be far from hygienic [26]. Persistent signs of sepsis require evaluation for the presence of endocarditis. Foreign bodies, such as broken needles, should be ruled out by radiography, and duplex sonography should be performed to identify the presence of vascular complications [27]. A broad-spectrum antibiotic effective against aerobic and anaerobic organisms should be administered in patients with these infections.

#### Infections developing in damaged skin

It is a heterogeneous group that includes soft tissue infections developing in damaged skin such as bite wounds (animal and human bites), burn wounds or in pressure or vascular ulcers. If managed incorrectly, these infections can develop into more complicated soft tissue infections

Soft tissue infection is the most common complication of animal and human bites [28–30]. The risk of infection depends on the type of bite, the site of injury, the time elapsed from the bite until presentation, host factors, and the management of the wound.

Antibiotic prophylaxis is always recommended for high-risk wounds. For patients with signs of sepsis, compromised immune status, severe comorbidities, associated severe cellulitis, severe and deep wounds, a broad-spectrum antibiotic effective against aerobic and anaerobic organisms is always required.

Patients with serious burn injury require immediate care. Significant burn injuries can predispose to infectious complications. Burn wound infections are one of the most important and potentially serious complications that occur in the acute period following injury. Accurate medications of the wound with early excision of the eschar can substantially decrease the incidence of invasive burn wound infection.

Although burn wound surfaces are sterile immediately following thermal injury, these wounds may be colonized with microorganisms. If the patient's host defenses and therapeutic measures (such as excision of necrotic tissue and wound medications) are inadequate, microorganisms can colonize viable tissue, and a burn wound infection can occur.

Burn wound infections usually are polymicrobial. They can be immediately colonized by gram-positive bacteria from the patient's endogenous skin flora or the external environment. However they can also be rapidly colonized by gram-negative bacteria, usually within a week of the burn injury.

Pressure ulcers are localized areas of tissue necrosis that tend to develop when soft tissue is compressed between a bony prominence and an external surface for a prolonged period of time. The damage may be relatively minor, or it may lead to massive destruction of deeper tissues. The majority of pressure ulcers develop in areas adjacent to the ischium, sacrum, and greater trochanter.

Combination of surgical and antimicrobial interventions may be required to manage infected decubitus ulcers. Surgical debridement is necessary to remove necrotic tissue. Antimicrobial therapy should be used for patients with severe pressure ulcer infections, including those with spreading cellulitis or patients with signs of sepsis. Because such infections usually are polymicrobial, therapeutic regimens should be directed against both gram-positive and gram-negative facultative organisms as well as anaerobic organisms.

In many cases a correct wound care management can largely prevent the appearance of these infections.

### Necrotizing soft tissue infections

NSTIs include necrotizing cellulitis, necrotizing fasciitis, Fournier’s gangrene and necrotizing myositis. They are life-threatening, invasive, soft tissue infections caused by aggressive, usually gas-forming bacteria. Delay in diagnosing and treating these infections increases the risk of mortality.

NSTIs may involve dermal and subcutaneous components (necrotizing cellulitis), fascial component (necrotizing fasciitis), and muscular components (necrotizing myositis) either singularly or in combination. NSTIs may also be classified into three types defined by the bacterial pathogens initiating the infection and their typical clinical characteristics; type 1 – poly-microbial, type 2 – mono-microbial pathogenic β-haemolytic Streptococci or CA-MRSA, type 3 – mono-microbial secondary to a variety of pathogenic bacilli. In the early phases, necrotizing soft tissue infections, cause localized inflammatory reactions in the involved tissues. Necrosis occurs because of direct cellular injury from bacterial endo/exotoxins [31], significant inflammatory oedema [32] within a closed tissue compartment, thrombosis of local blood vessels, and tissue ischemia. Circulating toxins may cause systemic illness [33] which can progress to septic shock, multisystem organ dysfunction, and death [34]. Patients with NSTIs due to either Staphylococcus aureus or group A Streptococcus (Streptococcus pyogenes) may develop toxic shock syndrome (TSS). TSS is a toxin-mediated acute life-threatening illness, where the systemically absorbed toxins act as super antigens, massively activating the host inflammatory response.

#### Necrotizing cellulitis

Necrotizing cellulitis is similar to non-necrotizing cellulitis in bacterial aetiology and pathogenesis but is more serious and may be rapidly progressive and accompanied by significant systemic inflammatory changes (toxic shock syndrome). The pathogenesis and severity of these infections are related to the particular pathogenicity of the strains of either β-haemolytic streptococci or CA-MRSA. In the presence of tissue necrosis, other bacteria may become secondarily involved, particularly anaerobes.

#### Necrotizing fasciitis

Necrotizing fasciitis (NF) is a NSTI involving the fascial planes overlying the muscle. Due to the tenuous blood and lymphatic supply of the fascia and the potential planes on either surface, infectious processes can spread rapidly and relatively unimpeded once initiated. Thus, NF comprises a spectrum of diseases characterized by extensive, rapidly progressive necrosis involving the fascia and peri-fascial planes and may secondarily involve the surrounding subcutaneous tissue, skin, and muscle [35].

NF is most often poly-microbial and synergistic in nature with the specific bacteria involved being related to the etiologic source or body region of origin.

This condition can involve any part of body but primarily involves extremities, abdomen or perineum. Anaya et al. in a study of 150 cases of necrotizing fasciitis reported that extremities were the most common site of infection (58%) followed by the abdomen and perineum [36].

Usually necrotizing fasciitis is precipitated by some form of injury or local pathological condition: blunt or penetrating trauma, surgical site infection, burns, ulcers, abscess and even child birth have been documented as precipitating factors for NF. Improperly treated superficial infections can progress to NF. Body piercing procedures and tattooing, very minor trauma such as abrasion and insect bites have also been found to be able to initiate NF. In some situations NF has arisen without an identifiable previous trauma or pathological condition [37].

The mortality associated with the disease is high and has been reported from 6% to as high as 76% [38]

#### Fournier’s gangrene

Fournier’s gangrene is a rapidly progressive, variant of necrotizing fasciitis involving the external genitalia and perineum. Due to the complexity of fascial planes, this infection may extend up to the abdominal wall, down into the thigh, into the perirectal and gluteal spaces, and occasionally, into the retroperitoneum.

It has a mortality rate that reaches the 20–50% in many contemporary series [39, 40].

Fournier’s gangrene is nearly universally poly-microbial in origin, and is often caused by aerobic and anaerobic gram positive and gram negative bacteria. The origin of the infection is identifiable in the majority of cases and is predominantly from anorectal, genito-urinary or local cutaneous sources [41].

Iatrogenic factors leading to Fournier's gangrene have been reported after injection sclerotherapy and banding of haemorrhoids, haemorrhoidectomy and stapled haemorrhoidopexy [42].

Diagnosis is based on clinical signs and physical examination. Imaging may be needed to confirm clinical suspicions and to help in identifying the extent of the soft tissue involvement, particularly in the peri-rectal and retroperitoneal planes.

Fournier’s Gangrene Severity Index (FGSI) is a standard score for predicting outcome in patients with Fournier gangrene and is obtained from a combination of physiological parameters at admission including temperature, heart rate, respiration rate, sodium, potassium, creatinine, leukocytes, haematocrit and bicarbonate. A FGSI score above 9 has been demonstrated to be sensitive and specific as a mortality predictor in patients with Fournier’s gangrene [43].

#### Necrotizing myositis

Necrotizing myositis is a rare NSTI that is a serious, life-threatening infection of the muscle with local and systemic complications [6]. These infections can progress rapidly due to the virulence of the etiologic pathogens.

Like other NSTIs, early and appropriate antimicrobial and surgical debridement are the cornerstones of management.

Healthy muscle tissue is usually quite resistant to infectious processes. However, muscle can become infected by specific virulent pathogens with significant exotoxin production or when muscle perfusion and viability are compromised. Necrotizing myositis and myonecrosis of healthy muscle may occur secondary to a variety of clostridial species and, less commonly, to virulent Strep. pyogenes. In these cases, the pathogenicity is believed to be related to exotoxins promoting thrombosis and break down healthy muscle. Both infections can arise spontaneously or secondary to injury or adjacent infection.

Myositis may also occur in muscle sites that have been compromised by injury, ischemia, malignancy or surgery. The predominant pathogens are S aureus, including community-associated MRSA, group A streptococci, gram-negative aerobic and facultative bacilli [44].

Presenting findings are localized pain in a single muscle group, muscle spasm, and fever.

### Principles of treatment

#### Antimicrobial therapy

The principal barrier against microbial invasion is the skin. It constantly interacts with the external environment and is colonized with different populations of bacteria. Intact and well vascularized skin is highly resistant to bacterial invasion.

The majority of SSTIs involving healthy skin are caused by aerobic Gram-positive cocci, specifically S. aureus and streptococci. Strains of S. aureus and Group A β-haemolytic streptococci (GAS) can produce a variety of toxins that may both potentiate their virulence and affect the soft tissues and allow invasion of the dermis [45].

Polymicrobial infections occur when aerobic Gram negative and anaerobes invade soft tissues.

SSTIs management has recently become more complicated because of the increasing prevalence of multidrug-resistant pathogens.

For SSTIs that develop following significant antibiotic exposure or in hospitals or other healthcare settings, the increasing resistance among both Gram-positive and Gram-negative bacteria makes empirical treatment regimens challenging.

Considerable variation in the resistance rates of *S. aureus* to methicillin (or oxacillin) has been noted between continents, with the highest rates in North America (35.9%), followed by Latin America (29.4%) and Europe (22.8%) [46].

Although MRSA has been usually acquired during exposure in hospitals and other healthcare facilities (HA-MRSA), there has been a recent increase in MRSA infections presenting in the community (CA-MRSA) [47].

CA-MRSA strains are genetically and phenotypically distinct from HA-MRSA. They may be susceptible to a wider range of anti-staphylococcal antimicrobials (some are resistant only to β-lactams). Populations at increased risk for CA-MRSA are listed below [47]:

- Children <2 years old
- Athletes (mainly contact-sport participants)
- Injection drug users
- Men who have sex with men
- Military personnel
- Inmates of correctional facilities, residential homes or shelters
- Vets, pet owners and pig farmers
- Patients with post-flu-like illness and/or severe pneumonia
- Patients with concurrent SSTI
- History of colonization or recent infection with CA-MRSA
- History of antibiotic consumption in the previous year, particularly quinolones or macrolides

CA-MRSA infections are becoming increasingly common. They can have a rapid and devastating course and may produce the pathogenic Panton–Valentine leucocidin toxin (PVL), which destroys white blood cells and is an important virulence factor [48, 49].

Antibiotics recommended for CA-MRSA infections are listed below.

**Outpatient treatment:**

- Minocycline100 mg q12h
- or
- Trimethoprim and Sulfamethoxazole 160/800 mg q12h
- or
- Doxycycline 100 mg q12h
- or
- Clindamycin 300–600 mg q8h
- or
- Linezolid 600 mg q12h

**Inpatient treatment:**

- Vancomycin 15 mg/kg IV q12h
- or
- Teicoplanin LD 12 mg/kg IVq h12h for 3 doses then 6 mg/kg q12 h
- or
- Tigecycline 100 mg IV as a single dose; then 50 mg IV q12h
- or
- Linezolid 600 mg q12h
- or
- Daptomycin 4–6 mg/kg q24h

For empirical coverage of CA-MRSA in outpatients with SSTI, oral antibiotic options include clindamycin, trimethoprim-sulfamethoxazole (TMP-SMX), a tetracycline (doxycycline or minocycline), and linezolid. If coverage for both β-hemolytic streptococci and CA-MRSA is required, options include clindamycin alone or TMP-SMX or a tetracycline in association with a β-lactam (eg, amoxicillin) or linezolid alone [50].

For hospitalized patients with severe SSTI, in addition to surgical debridement and broad-spectrum antibiotics, empirical therapy for MRSA should be considered, pending culture data. Options include intravenous (IV) vancomycin, PO or IV linezolid 600 mg twice daily, daptomycin 4 mg/kg/dose IV once daily, clindamycin 600 mg IV or PO 3 times a day [50], tigecycline 100 mg IV LD, then 50 mg twice daily.

Glycopeptides have been for many years the microbiological agents of choice for difficult Gram positive infections. Fortunately, staphylococcal resistance to glycopeptides remains rare, although rising minimal inhibitory concentrations (MICs) of glycopeptides may affect the efficacy of these antibiotics [51, 52].

Increased resistance to glycopeptides has encouraged the development of new agents active against Gram-positive bacteria, particularly for severe soft tissue infections where an aggressive antimicrobial management is always recommended, such as linezolid [53–55], tigecycline [56, 57] and daptomycin [58, 59].

Linezolid has been considered an agent of choice in complicated skin and soft-tissue infections (cSSTIs), It has the advantages of early intravenous-to-oral switch with the oral preparation having very high bioavailability and excellent tissue penetration [53, 54].

In 2010 an open-label study compared oral or intravenous linezolid with intravenous vancomycin for treatment of cSSTIs caused by MRSA.

Patients receiving linezolid had a significantly shorter length of stay and duration of intravenous therapy than patients receiving vancomycin. Both agents were well tolerated. Adverse events were similar to each drug's established safety profile [54].

Recently a Cochrane meta-analysis including all randomised controlled trials (RCTs) comparing linezolid with vancomycin in the treatment of SSTIs [55].

Linezolid was associated with a significantly better clinical (RR 1.09, 95% CI 1.03 to 1.16) and microbiological cure rate in adults (RR 1.08, 95% CI 1.01 to 1.16). For those infections due to MRSA, linezolid was significantly more effective than vancomycin in clinical (RR 1.09, 95% CI 1.03 to 1.17) and microbiological cure rates (RR 1.17, 95% CI 1.04 to 1.32).

The daily cost of outpatient therapy was less with oral linezolid than with intravenous vancomycin. Although inpatient treatment with linezolid cost more than inpatient treatment with vancomycin per day, the median length of hospital stay was three days shorter with linezolid [55].

Tigecycline has a broader range of activity, covering infections caused not only by resistant Gram-positive bacteria but also by many resistant Gram-negative organisms including those producing extended spectrum β-lactamases. It is only available as an intravenous formulation [56].

An interesting study evaluated the penetration of tigecycline into soft tissues in patients with cSSTIs requiring surgical intervention was published in 2011.

Tissue and blood samples were obtained one to six days (mean 2.5 days) after initiation of tigecycline treatment. The authors found higher concentrations of tigecycline in soft tissue than in the serum at the same time point [57].

Daptomycin has proven efficacy in patients with Gram-positive cSSTIs, including those caused by Staphylococcus aureus resistant to Methicillin [58].

Daptomycin has been shown to achieve very good concentrations in skin and soft tissues. In 2010 a meta-analysis compared effectiveness and toxicity of daptomycin with that of other antimicrobials for the treatment of SSTIs. Four studies were included in the analysis (3 were randomized RCTs). Vancomycin and semisynthetic penicillins were used in the comparator arm. Three studies reported on patients with cSSTIs. Daptomycin tissue penetration supports its use in the treatment of cSSTIs and it has been shown to be non-inferior to vancomycin and semi-synthetic penicillins [59].

Broad-spectrum antimicrobial therapy should be initiated as soon as possible. In a study of 492 patients with community-onset MRSA SSTIs, 95% of episodes treated with an active antibiotic within 48 hours were treated successfully, compared with an 87% rate of successful treatment in patients who did not receive an active antibiotic. After logistic regression analysis, failure to initiate active antimicrobial therapy within 48 hours of presentation was the only independent predictor of treatment failure (adjusted odds ratio [OR], 2.80; 95% CI, 1.26 to 6.22) [60].

Using data from more than 100 hospitals in the United States, Berger et al. [61] recently published a retrospective analysis of all adults hospitalized for complicated skin and skin-structure infections over a 9-year period. The authors defined "initial therapy" as all parenteral antibiotics administered <24 hours of admission, and such therapy was assumed to have failed if the patient (1) received new antibiotic (s) subsequently (excluding similar/narrower spectrum antibiotics or those begun at discharge), or (2) underwent drainage/debridement/amputation >72 h after admission.

Initial treatment failure in patients hospitalized for complicated skin and skin-structure infections was associated with significantly worse clinical outcomes, longer hospital stays, and higher hospital charges than with successful initial treatment.

#### Source control

Source control represents a key component of success in the management of sepsis [62].

Source control for SSTIs includes drainage of infected fluids, debridement of infected soft tissues, removal of infected devices or foreign bodies. It should also include definite measures to correct any anatomic derangement resulting in ongoing microbial contamination and restoring optimal function [5]. Delay in source control for patients with NSTIs has been repeatedly associated with a greater mortality.

#### Supportive treatment for the critically ill patients

NSTIs can present with a fulminant course and may be associated with great morbidity and high case-fatality rates, especially when they occur in conjunction with toxic shock syndrome.

Aggressive treatment of the underlying organ dysfunction is an essential component of improving patient outcome. Physiologic support including close invasive monitoring in an intensive care unit setting and aggressive resuscitation are often necessary in necrotizing infections.

#### Soft tissue infections in immunocompromised patients

SSTIs in immunocompromised patients can be challenging as they can be caused by unusual microorganisms. SSTIs in immunocompromised patients may progress rapidly, then becoming life threatening and are more difficult to eradicate with antibiotics alone, in the absence of an intact immune system [63]. Additionally, fungal SSTI infections are more common in immunocompromised populations.

There are few studies on immunocompromised patients with soft-tissue infections.

A single-institution retrospective cohort study evaluating immunocompromised patients with necrotizing soft tissue infections was recently published [63].

In this study immunodeficiency was defined by corticosteroid use, active malignancy, receiving chemotherapy or radiation therapy, diagnosis of human immunodeficiency virus or AIDS, or prior solid organ or bone marrow transplantation with receipt of chronic immunosuppression.

Immunocompromised patients with NSTIs were associated with delays in diagnosis and surgical treatment and with higher in-hospital mortality. At presentation, immunocompromised patients failed to exhibit typical clinical and laboratory signs of NSTIs. The authors concluded that physicians caring for such patient populations should maintain a higher level of suspicion for necrotizing soft tissue infections and consider early surgical evaluation and treatment.

### Recommendations

#### Surgical site infections

**1) Surgical site infections require prompt and wide opening of the surgical incision. Antimicrobial therapy is recommended for deep incisional surgical site infections if systemic signs of sepsis are present, if source control is incomplete or in immunocompromised patients (Recommendation 1 C).**

**2) In patients who have had clean operations, antimicrobial therapy should cover gram-positive organisms; in contrast, in patients who have had procedures on the gastrointestinal or genitourinary tract antimicrobial therapy should cover both gram-positive and gram-negative organisms (Recommendation 1 C).**

Surgical site infections are caused by a variety of organisms.

The pathogens isolated from infections differ, primarily depending on the type of surgical procedure. In clean surgical procedures, in which the gastrointestinal, gynecologic, and respiratory tracts have not been entered, Staphylococcus aureus from the exogenous environment or the patient's skin flora is the usual cause of infection. In other categories of surgical procedures, including clean-contaminated, contaminated, and dirty, the polymicrobial aerobic and anaerobic flora closely resembling the normal endogenous microflora of the surgically resected organ are the most frequently isolated pathogens [4].

Treatment involves widely opening the incision. Antimicrobial therapy is required in deep infections if systemic signs of toxicity are present, if source control is incomplete or in immunocompromised patients. Broad-spectrum empiric antimicrobial therapy should initially be selected to cover potentially resistant pathogens. Cultures should be always obtained and antimicrobial therapy modified based on the culture and sensitivity results.

#### Non necrotizing superficial infections

**3) Erysipelas, and cellulitis are managed by antimicrobial therapy against Gram-positive bacteria (Recommendation 1 C)**.

**4) Lack of clinical response for cellulitis may be due to resistant strains of staphylococcus or streptococcus, or deeper processes, such as necrotizing fasciitis or myonecrosis (Recommendation 1 C).**

**5) Therapy for community-acquired MRSA should be recommended for patients at risk for CA-MRSA or who do not respond to beta-lactam therapy within 48 to 72 hours (Recommendation 1 C).**

**6) If a streptococcal toxic shock syndrome is suspected, an antiribosomal agent such as clindamycin or linezolid can be used to reduce exotoxin and superantigen production (Recommendation 1 C).**

The term “non-necrotizing cellulitis” has been used to incorporate two entities, erysipelas and cellulitis, which are diffusely spreading skin infections not associated with suppurative foci.

However, a fine distinction exists between erysipelas and cellulitis. Erysipelas has a clear line of demarcation between involved and uninvolved tissue and lesions raised above the surrounding normal skin [64].

Cellulitis involves deeper layers of the dermis and subcutaneous tissue and has less distinctive features than erysipelas [64].

They are commonly caused by streptococcal species, usually b-hemolytic streptococci (usually group A). S. Aureus rarely causes erysipelas and cellulitis [64], but such infections usually are more suppurative and less diffuse.

Superficial, non-necrotizing infections caused by certain strains of Group A-streptococcal (GAS) may be associated with streptococcal toxic shock syndrome, characterized by rapid progression of septic shock and organ failure [65].

As previously discussed, the recent dramatic increase in CA-MRSA makes the empiric treatment of staphylococcal infections troubling.

Diagnostic studies have a low yield in patients with superficial soft tissue infections and usually do not help with the diagnosis. However, establishing whether cellulitis represents signs of a deeper and/or more severe infectious process can be difficult but is of considerable importance as the majority of NSTIs are originally diagnosed as cellulitis.

Lack of clinical response could be due to unusual organisms, resistant strains of staphylococcus or streptococcus, or deeper processes, such as necrotizing fasciitis. In patients who become increasingly ill, deeper infections should be always suspected.

For these infections, a penicillinase-resistant penicillin is the drug of choice.

Moxifloxacin or Levofloxacin may be suggested in patients who are allergic to beta-lactams. Clindamycin reduces exotoxin and superantigen production by pathogenic strains of GAS, and can be used in association as an adjunct in the treatment of streptococcal TSS [66].

Therapy for community-acquired MRSA should be added for patients at risk for CA-MRSA and subsequently for patients who do not respond to beta-lactam therapy within 48 to 72 hours or who have chills, fever, a new abscess, increasing erythema, or uncontrolled pain [67].

Although in cellulitis, swabs and aspirates of the leading edge of the site of infection have a low yield of around 10% [68], when it is possible, antibiotic treatment should be modified, on the basis of antimicrobial susceptibilities of organisms obtained.

#### Simple abscesses

**7) Incision and drainage is the primary treatment for a simple abscesses or boils. Antibiotics are not needed (Recommendation 1 C).**

For a simple superficial abscess or boil, incision and drainage is the primary treatment, and antibiotics are not needed [21, 22]. Simple abscesses should not have extension into deeper tissues or multiloculated extension.

#### Complex abscesses

**8) Complex skin and subcutaneous abscesses are typically well circumscribed and respond to incision and drainage. Antimicrobial therapy is required if systemic signs of sepsis are present, in immunocompromised patients, if source control is incomplete or for the abscesses with significant cellulitis (Recommendation 1 C).**

**9) Empiric antibiotic therapy should be directed toward the likely pathogens involved.**

**Therapy for community-acquired MRSA should be recommended for patients at risk for CA-MRSA (Recommendation 1 C).**

**10) Inadequate resolution should prompt consideration of further drainage, resistant pathogens, or host immune failure (Recommendation 1 C).**

The cornerstone of treatment is early surgical drainage. Antimicrobial therapy is required if systemic signs of sepsis are present, in immunocompromised patients, if source control is incomplete or for the abscesses with significant cellulitis.

Complicated abscesses may involve a variety of pathogens. Aerobic gram-positive pathogens are isolated in most complicated abscesses. They may involve only a single pathogen but are frequently poly-microbial in origin and may involve a number of organisms [69].

Empiric antibiotic therapy should be directed toward the likely pathogens involved. Broad-spectrum agents with coverage of gram-positive, gram-negative, and anaerobic pathogens may be required depending on the clinical setting. Given the high frequency of MRSA, this pathogen should be empirically covered [1] if it is suspected, but no randomized studies are available for the treatment of SSTI specifically caused by CA-MRSA.

#### Diagnosis of necrotizing soft tissue infections

**11) A rapidly progressive soft tissue infection should be always treated aggressively as a necrotizing soft tissue infection (Recommendation 1 C).**

Initially, distinguishing between cellulitis and a necrotizing soft tissue infection that requires operative intervention may be difficult. Most cases of necrotizing soft tissue infection are originally diagnosed as cellulitis. However, since time to operative debridement is a strong determinant of outcome in NSTIs, timely diagnosis is essential.

Patients with necrotizing soft tissue infections usually present with severe pain that is out of proportion to the physical findings.

Necrotizing infections more commonly present with systemic signs of sepsis than non-necrotizing infections, although TSS from both S. aureus and Strep. pyogenes can occur without a necrotizing process. Physical findings of necrotizing soft tissue infections may include tenderness beyond the area of erythema, crepitus, and cellulitis that is refractory to antimicrobial therapy.

A rapidly progressive soft tissue infection should be initially treated as a necrotizing infection. Bullous changes, ecchymotic changes of the skin, and loss of skin sensation should all be considered as a necrotizing infection. Crepitus is most commonly the result of gas within tissues. While the presence of crepitus and gas within tissues is highly suggestive of a NSTI, its absence does not rule out its presence. All bacteria growing under aerobic conditions produce CO2 as a by-product and the free diffusion of CO2 typically limits gas collection in these cases, thus explaining the absence of gas in rapidly progress S. pyogenes infections.

The clinical picture may worsen very quickly, sometimes during a few hours.

Delay in diagnosis and/or treatment correlates with a poor outcome, leading to severe sepsis and/or multiple organ failure and only early surgical debridement and appropriate antimicrobial treatment can prevent sepsis progression and death.

In order to predict the presence of necrotizing soft tissue infection, a group of investigators described the development and application of the Laboratory Risk Indicator for Necrotizing infection (LRINEC) score in a 2004 report. This scoring system assigned points for abnormalities in six independent variables: serum C-reactive protein level (>150 mg/L), WBC count (>15,000/μL), hemoglobin level (<13.5 g/dL), serum sodium level (<135 mmol/L), serum creatinine level (>1.6 mg/dL), and serum glucose level (>180 mg/dL). With a score of 8 or higher, there is a 75% risk of a necrotizing soft tissue infection. The authors recommended that the LRINEC score be used to determine which patients require further diagnostic testing, given that the negative predictive value of this screening tool was 96% [70]. Subsequent evaluation of the LRINEC score has demonstrated that it lacks the sensitivity to be a useful adjunct for the diagnosis of necrotizing infections.

The diagnosis of NSTIs is primarily a clinical diagnosis. However, plain radiographs, ultrasound, computed tomography (CT), magnetic resonance imaging (MRI), may be able to provide useful information for necrotizing infection when the diagnosis is uncertain [71].

The most common plain radiographic findings are similar to those of cellulitis, with increased soft-tissue thickness and opacity. Frequently, plain radiographs are normal unless the infection and necrosis are advanced. The characteristic finding is gas in the soft tissues, but subcutaneous gas may be present in few cases of necrotizing infection [72] and is not present in pure aerobic infections such as those caused by S. pyogenes.

Additionally, subcutaneous gas may not be present in earlier stages of the disease process and only become manifest as the patient’s condition deteriorates.

**12) Both CT and MRI may be useful for diagnosing necrotizing soft tissue infections. However MRI may be difficult to perform under emergency situations. (Recommendation 2 C).**

**13) In unstable patients ultrasound may be useful to differentiate simple cellulitis from necrotizing fasciitis (Recommendation 2 C).**

Computed tomography (CT) has a higher sensitivity than plain radiography in identifying early necrotizing fasciitis. Findings consistent with necrotizing fasciitis are fat stranding, fluid and gas collections that dissect along fascial planes, and gas in the involved soft tissues. Additionally, fascial thickening and non-enhancing fascia on contrast CT suggests fascial necrosis [73].

In 2010 a case series study [74] about CT for the diagnosis of necrotizing soft tissue infections was published. Of 67 patients with study inclusion criteria, 58 underwent surgical exploration, and necrotizing soft tissue infection was confirmed in 25 (43%). The remaining 42 patients had either non-necrotizing infections during surgical exploration (n = 33) or were treated non-operatively with successful resolution of the symptoms (n = 9). The sensitivity of CT to identify NSTI was 100%, specificity was 81%, positive predictive value was 76%, and negative predictive value was 100%.

Magnetic resonance imaging has been the imaging modality of choice for necrotizing fasciitis. The patients with NF have usually a significantly greater frequency of the following MR findings: thick (≥3 mm) abnormal signal intensity on fat-suppressed T2-weighted images, low signal intensity in the deep fascia on fat-suppressed T2-weighted images, a focal or diffuse non-enhancing portion in the area of abnormal signal intensity in the deep fascia, extensive involvement of the deep fascia, and involvement of three or more compartments in one extremity [75].

Schmid et al. found MRI to be 100% sensitive and 86% specific and have a diagnostic accuracy of 94% for diagnosing necrotizing fasciitis. However, the authors noted that MRI imaging tends to overestimate the extent of deep fascial involvement. Therefore, treatment should be based on a combination of clinical findings and MRI results [76].

Ultrasound has the advantage of being rapidly performed at bedside and may be helpful in differentiating simple cellulitis from necrotizing fasciitis. In a prospective observational study of 62 patients with clinically suspected necrotizing fasciitis, ultrasound had a sensitivity of 88.2%, specificity of 93.3%, positive predictive value of 95.4%, negative predictive value of 95.4%, and diagnostic accuracy of 91.9%. The authors considered the findings of diffuse subcutaneous thickening accompanied with fluid accumulation of >4 mm in depth along the deep fascial layer predictive of necrotizing fasciitis [77].

Fascial biopsy with frozen section has been suggested as a means to achieve earlier diagnosis of NSTIs [78, 79]. However frozen biopsy is not very practical and requires availability and experience from the pathologists, and the time taken to carry out and analyze the sample could be used for debridement [80].

The Finger test is another adjunct method described for diagnosing NSTIs. It is performed under local anesthesia. A 2-cm incision is made down to the deep fascia. A minimal tissue resistance to finger dissection (positive Finger test), the absence of bleeding, presence of necrotic tissue, and/or murky and grayish (“dishwater”) fluid following incision, all may support the diagnosis of NSTI [81].

#### Management of necrotizing soft tissue infections

**14) Patients with severe sepsis or septic shock caused by NSTI require early source control, antimicrobial therapy, and supportive treatment (Recommendation 1 C).**

#### Source control of necrotizing soft tissue infections

**15) Surgical source control must be early and aggressive to halt progression of the inflammatory process caused by NSTI (Recommendation 1 C).**

The most critical factors for reducing mortality from necrotizing soft tissue infections are early recognition and urgent operative debridement [82].

Surgical debridement must be aggressive to halt progression of infection. Cultures of infected fluid and tissues should be obtained during the initial surgical debridement and the results used to tailor specific antibiotic management.

Radical surgical debridement of the entire affected area should be performed, continuing the debridement into the healthy-looking tissue.

In the setting of Fournier’s gangrene, diverting colostomy has been demonstrated to improve the outcome and the need for fecal diversion depends upon severity of the disease. It helps in decreasing sepsis by minimizing bacterial load in the perineal wound thus controlling infection [83].

Diverting colostomy does not eliminate the necessity of multiple debridements, nor reduces the number of these procedures [84].

Recently rectal diversion devices have been marketed. They are silicone catheters designed to divert fecal matter in patients with diarrhea, local burns, or skin ulcers. The devices protect the wounds from fecal contamination and reduce in the same way a colostomy does, both the risk of skin breakdown and repeated inoculation with colonic microbial flora. Estrada et al. showed that it was effective way for fecal diversion and forms an alternative to colostomy [85].

Postoperative wound care starts with meticulous haemostasis. Non-adherent compressive dressings should be applied, followed by repeat wound inspection in ≤ 24 hours [86].

Any patient with extensive necrosis or who is considered to have not be adequately debrided at the initial operation should be returned to the operating room in 24–48 hours for a second look [5].

Further debridement should be repeated until the infection is controlled.

Several reports have documented the utility of vacuum-assisted wound closure (VAC) therapy for managing patients who have acute NSTI demonstrating that VAC technique of wound closure is effective in managing non-healing limb wounds consequential to surgical treatment for patients suffering from acute necrotizing fasciitis [87–89]. Negative pressure therapies should be reserved for use only after adequate source control has been obtained.

#### Hyperbaric oxygen therapy

**16) Although the benefit of adjuvant hyperbaric oxygen (HBO) therapy remains controversial, it may be considered where it is available (Recommendation 2 C).**

The role of hyperbaric oxygen (HBO) as an adjunctive treatment is still controversial, and no prospective randomized clinical trials have been published. There is very little evidence supporting the benefits of hyperbaric oxygen therapy in treating NSTI.

In 2009, a retrospective review investigating the effect of HBO in treating NSTI was published [90].

Adjunctive use of HBO to treat necrotizing soft tissue infections did not reduce the mortality rate, number of debridements, hospital length of stay, or duration of antibiotic use.

In order to determine the effect of hyperbaric oxygen HBO therapy on mortality, complication rate, discharge status/location, hospital length of stay and inflation-adjusted hospitalisation cost in patients with NSTIs a retrospective study of 45,913 patients in the Nationwide Inpatient Sample from 1988 to 2009 was published in 2012 [91]. This retrospective analysis of HBO therapy in NSTI showed that despite the higher hospitalisation cost and longer length of stay, the statistically significant reduction in mortality supports the use of HBO therapy in NSTI.

Recently a review about HBO therapy for treating acute surgical and traumatic wounds was published [92]. The authors concluded that there is a lack of high quality, valid research evidence regarding the effects of HBO therapy on wound healing.

Although there is a trend in clinical outcomes which shows that HBO therapy may be useful in managing NSTI [93], the benefit of adjuvant HBO therapy for NSTI remains controversial, and more robust evidence by prospective randomized trials is necessary. HBO therapy should always be considered as an adjunct treatment and should never replace surgical debridement.

The expert panel supports the use of HBO therapy in those hospitals where the hyperbaric chamber is available**.**

#### Antimicrobial therapy

**17) Early appropriate empiric coverage against suspected pathogens should be initiated, based upon the clinical setting for patients with NSTI (Recommendation 1 C).**

**18) Patients whose clinical setting or gram stain suggests rapidly progressive infection potentiated by exotoxins from Gram positive pathogens (S. pyogenes, CA-MRSA, Clostridial species), treatment with antimicrobial agents should be combined with antiribosomal agents (clindamycin or linezolid). Patients who present with rapidly progressive infections with gram stains of tissue demonstrating gram negative pathogens (Aeromonas sp., Eikenella, Vibrio sp) should be treated with antiribosomal agents targeting gram negative pathogens (tetracyclines) (Recommendation 1 C).**

**19) Appropriate empiric coverage against MRSA should be immediately initiated in patients with necrotizing soft tissue infection (Recommendation 1 C).**

**20) Since it is impossible to exclude with certainty a polymicrobial necrotizing infection, an aggressive broad-spectrum empiric antimicrobial therapy should initially be selected to cover gram-positive, gram-negative, and anaerobic organisms until culture-specific results and sensitivities are available (Recommendation 1 C).**

**21) An appropriate de-escalation of antimicrobial therapy is suggested once culture results return (Recommendation 1 C).**

Microbiologically, NSTIs have been classified as either type 1 (polymicrobial) or type 2 (mono-microbial) or type 3 (mono-microbial infections initiated by a variety of virulent gram positive or gram negative bacilli such as Clostridia, Vibrio, Aeromonas, Eikenella, and Bacillus species) [80, 94, 95]. Occasionally in immunocompromised patients NSTIs may be also caused by mycotic species.

Polymicrobial infections are more common, with cultures yielding a mixture of aerobic and anaerobic organisms. These infections typically occur in the perineum and trunk.

NF is associated with surgical procedures involving the bowel [96] or penetrating abdominal trauma, decubitus ulcer, perianal abscess, the site of injection in intravenous drug users, and spread from a perineal or vulvo-vaginal infection [6]. The etiologic isolates consist of Gram-positive organisms, such as Staphylococcus aureus, S. pyogenes, and enterococci; Gram-negative aerobes, such as Escherichia coli; and anaerobic organisms, such as Bacteroides or Clostridia species.

Mono-microbial infections are less common than the poly-microbial variety. These typically occur in the limbs and afflict healthy patients with no implicative comorbidities. There may be often a history of trauma, frequently trivial.

As S. pyogenes and S. aureus and Group A Streptococcus are the usual pathogens [97], selection of specific antimicrobials that inhibit toxin production by may be helpful, particularly in those patients who have evidence of toxic shock syndrome [98].

An acceptable empiric antimicrobial regimen should always include antibiotics, which covers CA-MRSA with the additional benefit of inhibiting invasive group A Streptococcus virulence proteins.

Selection of antibiotics that inhibit toxin production may be helpful, particularly in those patients who have evidence of toxic shock syndrome, potentially present in patients who have streptococcal and staphylococcal infections. Protein cytotoxins play an important role in the pathogenesis of various staphylococcal infections, and toxin production should be considered when selecting an antimicrobial agent for gram-positive pathogens [99]. Linezolid and clindamycin plays an important role because it may significantly reduce the early release of exotoxins from Gram positive pathogens [99, 100].

Since it is impossible to exclude with certainty a poly-microbial infection, an aggressive broad-spectrum empiric antimicrobial therapy should initially be selected to cover gram-positive, gram-negative, and anaerobic organisms until culture-specific results and sensitivities are available.

Empiric antimicrobial therapy should be started as soon as possible.

Subsequent modification (de-escalation) of the initial regimen becomes possible later, when culture results are available and clinical status can be better assessed, 24–72 hours after initiation of empiric therapy.

In Appendix 1 antimicrobial regimens for necrotizing soft tissue infections are listed.

#### Supportive treatment

**22) Supportive treatment in managing NSTI must be early and aggressive to halt progression of the inflammatory process (Recommendation 1 A).**

Early detection of severe sepsis and prompt aggressive treatment of the underlying organ dysfunction is an essential component of improving outcome of critical ill patients.

Deep soft tissue infections may present with a fulminant course and may be associated with great morbidity and high case-fatality rates, especially when they occur in conjunction with toxic shock syndrome.

After initial debridement, and early antimicrobial therapy, patients require early intensive care for haemodynamic and metabolic support. Patients may loss fluids, proteins and electrolytes from a large surgical wound [101]. In addition hypotension is caused by vasodilation induced by the systemic inflammatory response syndrome to infection [102].

Fluid resuscitation and analgesia are the mainstays of support for patients with advanced sepsis usually combined with vasoactive amines associated with mechanic ventilation.

These patients often exhibit extensive extracellular fluid sequestration within the affected area, as well as more generalized sequestration resulting from sepsis. The adequacy of intravascular volume repletion is assessed by mean arterial pressure (MAP) >65 mm Hg, central venous pressure (CVP) of 8–12 mmHg in combination with a central venous oxygen saturation (ScvO2) > 70% and Urine output >0.5 mL/kg/hr [103].

It has been established that the general prognostic value of a lactate of 4 mM/L on hospital admission is important; multiple studies have confirmed the risk stratification of this lactate level for illness severity and mortality in both the pre-hospital and in-hospital setting [104–108]. Lactate clearance has also been associated with decreased mortality in patients with severe sepsis and septic shock [109].

The absence of clear benefits following the administration of colloid solutions compared to crystalloid [110], supports a high-grade recommendation for the use of crystalloid solutions in the initial resuscitation of patients with severe sepsis and septic shock [103].

In patients whose hypotension does not resolve with appropriate intravascular fluid resuscitation, vasopressor agents are useful for raising blood pressure, improving myocardial function, and increasing organ and tissue perfusion [103].

#### Intravenous immunoglobulins

**23) Intravenous immunoglobulins may be considered in all patients with NSTI and evidence of organ dysfunction (Recommendation 2 C).**

The use of intravenous immunoglobulin for treating necrotizing soft tissue infections remains controversial, but is based on a potential benefit related to binding of gram-positive organism exotoxins [111].

Intravenous immunoglobulin therapy has been advocated for NSTI caused by streptococci and staphylococci. Intravenous immunoglobulin provides antibodies that can neutralize circulating exotoxins produced by these organisms and may modulate the systemic inflammatory response induced by cytokine stimulation [112].

Expert panel supports the use of early immunoglobulins in all the patients with NSTI associated to severe sepsis and septic shock.

#### Nutritional support in patients with necrotizing soft tissue infection

**24) Early nutritional support should be established (Recommendation 2C).**

Adequate nutritional support improves outcomes [113]. The best and simple assessment of prior nutritional state is a detailed history of prior illness and nutritional intake combined with clinical examination of fat and muscle distribution. The enormous endocrine and cytokine burst of systemic inflammatory response common to necrotizing infection will increase basal metabolic rate and nutritional requirements. Currently recommendations suggest that 25 kcal/kg/day is a reasonable target intake for ICU patients initially for the first week however it may be inadequate in the long run and a target of 30 or 35 kcal/kg/day [114–116].

There are no data showing improvement in relevant outcome parameters using early enteral nutrition (EN) in necrotizing soft tissue infection patients.

Parenteral nutrition (PN) should be reserved for patients in whom EN is contraindicated or is unlikely to meet nutritional requirements within 4 to 5 days.

## Appendix 1

### Antimicrobial therapy for necrotizing soft tissue infections

#### Necrotizing fasciitis

Linezolid 600 mg bid

- +
- Piperacillin/tazobactam 4/0-5 g LD infused in 30’ then 16/2 g qd by CI
- Or
- Daptomycin 6 mg/kg qd
- +
- Piperacillin/tazobactam 4/0-5 g LD infused in 30’ then 16/2 g qd by CI
- +
- Clindamycin 600–900 mg qid

### Fournier’s gangrene

*If signs and symptoms of severe sepsis are not present*

- Piperacillin/tazobactam 4/0-5 g LD infused in 30’ then 16/2 g qd by CI
- +
- Clindamycin 600–900 mg qid
- *If signs and symptoms of severe sepsis are present*
- Meropenem 1 g LD infused in 30’ then 1 g qid by extended infusion (3 to 6 h)
- +
- Linezolid 600 mg bid

### Necrotizing cellulitis

*If signs and symptoms of severe sepsis are not present*

- Amoxicillin/Clavulanate 2/0.2 gm qid IV
- +
- Clindamycin 600 mg qid IV
- *If signs and symptoms of severe sepsis are present*
- Linezolid 600 mg bid
- +
- Piperacillin/tazobactam 4/0-5 g LD infused in 30’ then 16/2 g qd by CI
- Or
- Daptomycin 6 mg/kg qd
- +
- Piperacillin/tazobactam 4/0-5 g LD infused in 30’ then 16/2 g qd by CI
- +
- Clindamycin 600–900 mg qid

### Necrotizing myositis

*If signs and symptoms of severe sepsis are not present*

- Amoxicillin/Clavulanate 2/0.2gm qid IV
- +
- Clindamycin 600 mg qid IV
- *If signs and symptoms of severe sepsis are present*
- Linezolid 600 mg bid
- +
- Piperacillin/tazobactam 4/0-5 g LD infused in 30’ then 16/2 g qd by CI
- Or
- Daptomycin 6 mg/kg qd
- +
- Piperacillin/tazobactam 4/0-5 g LD infused in 30’ then 16/2 g qd by CI
- +
- Clindamycin 600–900 mg qid
